# Synthesis of Structured Lipids by Lipase-Catalyzed Interesterification of Triacetin with Camellia Oil Methyl Esters and Preliminary Evaluation of their Plasma Lipid-Lowering Effect in Mice

**DOI:** 10.3390/molecules18043733

**Published:** 2013-03-25

**Authors:** Yu Cao, Suijian Qi, Yang Zhang, Xiaoning Wang, Bo Yang, Yonghua Wang

**Affiliations:** 1 School of Bioscience and Biotechnology, South China University of Technology, Guangzhou 510006, China; 2 College of Light Industry and Food Sciences, Key Lab of Fermentation and Enzyme Engineering, South China University of Technology, Guangzhou 510640, China

**Keywords:** structured lipids, lipase, fasting test, interesterification, triacylglycerol

## Abstract

Structured lipids (SLCTs triacylglycerols with short- and long-chain acyl residues) were synthesized by interesterification of triacetin and fatty acid methyl esters (FAMEs) from camellia oil, followed by molecular distillation for purification. Different commercial immobilized lipases (Lipozyme RM IM and Novozyme 435), the substrate molar ratios of FAMEs to triacetin, the reaction temperatures and the lipase amounts were studied for their efficiency in producing SLCTs. Results showed that Novozyme 435 was more suitable for this reaction system. Moreover, the optimal reaction conditions for the highest conversion of FAMEs and the highest LLS-TAGs (triacylglycerols with one short- and two long-chain acyl residues) yields were achieved at a molar ratio of FAMEs to triacetin of 3:1, 50 °C of reaction temperature and a lipase amount of 4% (w/v). Scale-up was conducted based on the optimized reaction conditions. Results showed that after 24 h of reaction , the conversion rate of FAMEs was 82.4% and the rate of disubstituted triacetin was 52.4 mol%. The final product yield rate was 94.6%. The effects of the synthesized SLCTs on the plasma lipid level of fasting mice were also studied. The SLCTs could effectively lessen the total triacylglycerol levels in plasma compared to the triacylglycerol group in fasting NIH mice. It suggested that this type of structured lipid might be beneficial for human health, especially for the prevention of obesity.

## 1. Introduction

Low-calorie structured lipids (SLs) are tailor-made triacylglycerols (TAGs) that are mainly designed for special nutritional applications, especially to meet for the growing need for healthier foods and to prevent obesity [1,2]. Nowadays, the most familiar types of low-calorie lipids are triacylglycerols with short- and long-chain acyl residues (SLCTs), triacylglycerols with medium- and long-chain acyl residues (MLCTs) and diacylglycerols (DAGs) [3]. To act as an ideal lipid substitute, the products should contain unsaturated fatty acids, especially essential fatty acids, and have no harmful effects. However, most of the reported SLs and DAGs products either have no unsaturated fatty acids or have potential safety problems. For example, Salatrim, one of the commercialized SLCTs products, was produced mainly by lipase-catalyzed with hydrogenated oil, which is potentially harmful, and triacylglycerols with only short-chain acyl residues (SCTs) by interesterification [4,5]. Some other SLCTs were produced by transesterification between TAGs and saturated fatty acids [6,7] or by interesterification of tributyrin and methyl stearate [8]. The resulting SLs products therefore lacked unsaturated fatty acids. DAGs were found very effective in reducing blood lipid level and body weight [9]. However, glycidyl esters were readily formed by intermolecular elimination of a fatty acid from DAGs due to the free acetoxyl group present [10]. Considering that the glycidyl esters might be hydrolyzed to the harmful parent glycidol by lipases in the gastrointestinal tract, DAGs products face safety problems [11].

In this study, a novel SLCT was designed and synthesized based on lipase-catalyzed interesterification of camellia oil FAMEs and triacetin. The SLCT product contains relatively high amounts of unsaturated fatty acids and has a lower risk of safety problems. The novel SLCT has a similar structure to DAG, but does not contain the free acetoxyl moiety. Camellia oil is an important cooking oil in China, which is comparative to olive oil [12]. It contains a large percentage of monounsaturated fatty acids (MUFAs) which are beneficial to health [13,14]. Triacetin was also found to be metabolically beneficial in hypermetabolic states [15], and effective in lowering the risk of phlebitis and thrombosis [16]. Moreover, FAMEs have lower melting point, which would avoid the bad effect of high temperature on enzymatic activity. The interesterification of FAMEs and triacetin is conducive to the purification of the final synthetic product by molecular distillation. FAMEs, triacetin and LSS-TAGs (triacylglycerols with two short- and one long-chain acyl residues) would be removed easily.

During interesterification, acyl migration happens easily due to the existence of MAGs (monoglycerols) and DAGs (diacylglycerols), resulting in the formation of LLL-TAGs (triacylglycerols with three long-chain acyl residues) as an impurity [17]. In this study, acyl migration could not be completely avoided, but it could be decreased to a relatively low level. The objective of this study was to optimize the synthesis of the novel SL, emphasising on high LLS-TAGs yielding, high conversion rate of FAMEs and low content of LLL-TAGs. The product was purified by molecular distillation, and its plasma lipid-lowering effects were also studied by fasting experiments in NIH mice.

## 2. Results and Discussion

### 2.1. Comparison of Lipase

**Figure 1:**
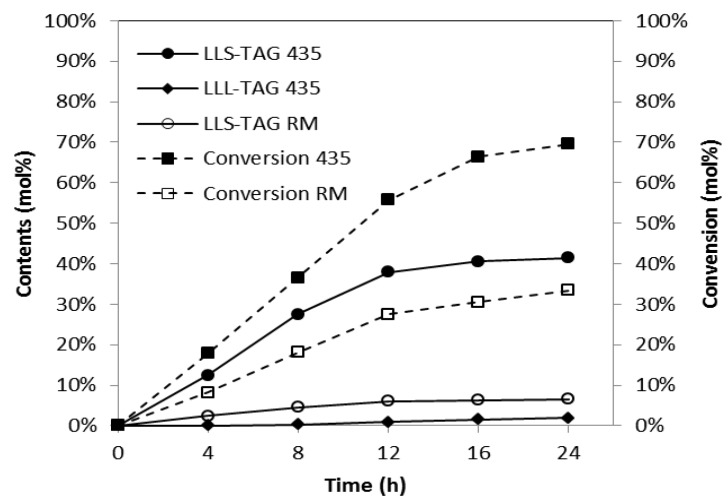
Efficiency of different lipases for the conversion of SLCTs at different time points. Reaction conditions were as follows: substrate molar ratio of FAMEs to triacetin: 4:1; lipase loading: 2% (w/w); temperature: 40 °C. LLS-TAG 435, the content of LLS-TAGs catalysis by Novozyme 435; LLL-TAG 435, the content of LLL-TAGs catalysis by Novozyme 435; LLS-TAG RM, the content of LLS-TAGs catalysis by Lipozyme RM IM; Conversion 435, the conversion of FAMEs catalysis by Novozyme 435; Conversion RM, the conversion of FAMEs catalysis by Lipozyme RM IM.

Two commercial immobilized lipases: *Rhizomucor miehei* (Lipozyme RM IM) and *Candida antarctica* (Novozyme 435) were compared for their efficiency in producing SLCTs by interesterification. As shown in Figure 1, the content of LLS-TAGs was 41.47%, and the conversion of FAMEs was 69.5% at 24 h with the use of Novozyme 435. The values were much higher than those obtained from Lipozyme RMIM, which were 6.54% and 33.4%, respectively. LLL-TAGs began to occur in the reaction catalyzed by Novozyme 435 from 12 h, and reached 1.54% at 24 h. As a whole, Novozyme 435 displayed a stronger activity in the reaction of triacetin than Lipozyme RM IM. It had been reported that Lipozyme RM IM had low catalysis activity with acetic acid [18]. Therefore, Novozyme 435 was selected for the subsequent studies.

### 2.2. Effects of Substrate Molar Ratio

**Figure 2:**
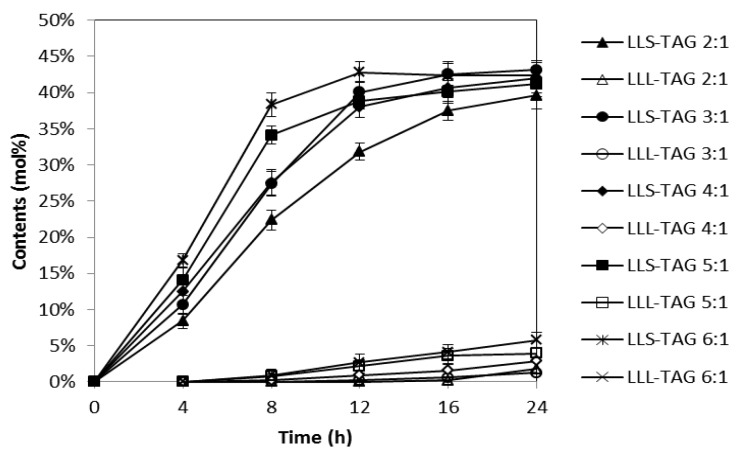
Effect of substrate molar ratio on the production of SLCTs. The reaction mixture containing different molar ratios of FAMEs/triacetin and 2% (w/w) Novozyme 435 was incubated at 40 °C. LLS-TAG 2:1, 3:1, 4:1, 5:1 and 6:1 the content of LLS-TAGs at molar ratio of FAMEs/triacetin from 2:1 to 6:1, respectively; LLL-TAG 2:1, 3:1, 4:1, 5:1 and 6:1 the content of LLL-TAGs at molar ratio of FAMEs/triacetin from 2:1 to 6:1, respectively.

The substrate molar ratio of FAMEs to triacetin was a crucial factor for the composition of final product during the interesterifications. To find out the optimal molar ratio of FAMEs to triacetin for high yields of LLS-TAGs yet low yields of LLL-TAGs at the same time, the interesterification was performed at 40 °C at various substrate molar ratios with 2% (w/w) Novozyme 435. Results are shown in Figure 2. The LLS-TAGs content increased with the increase of FAMEs in the substrate molar ratio. The highest content of LLS-TAGs, which was 45.2%, was achieved at the substrate molar ratio (FAMEs to triacetin) of 3:1. However, the content increase of LLS-TAGs became very slow once the molar ratio reached 4:1. The content of LLL-TAGs, which were from 1.8% to 5.8%, also increased with the increasing molar ratio of FAMEs to triacetin. The reason was that the higher amounts of FAMEs would elevate the reaction equilibrium and increase the ratio of collisions between substrates and enzyme. There there are two steps in interesterification. First, TAGs are hydrolyzed to MAGs and DAGs; second, new TAGs are synthesized by the esterification of acyl donors with MAGs and DAGs [17]. Larger amounts of FAMEs enhanced the opportunity for acyl migration, which could increase with time [19]. Therefore, the substrate mass ratio of FAMEs to triacetin was fixed at 3:1 in the following experiments for the production of highly pure LLS-TAGs.

### 2.3. Effects of Reaction Temperature

**Figure 3:**
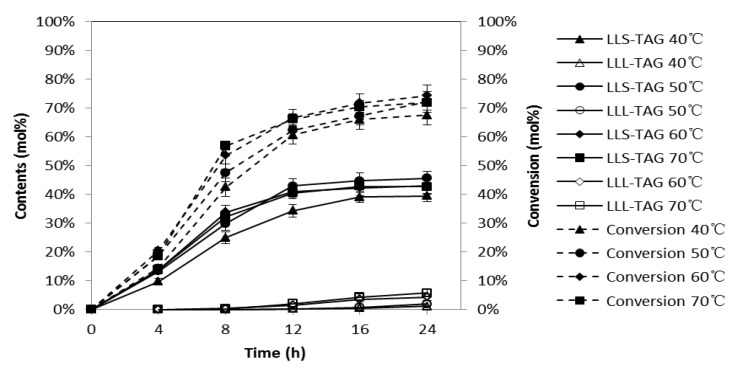
Effects of reaction temperature on interesterification of SLCTs at different time points. The reaction mixture was 9.7g FAME/triacetin (3:1 mol/mol) and 2% (w/w) Novozyme 435. LLS-TAG 40 °C, 50 °C, 60 °C and 70 °C, the content of LLS-TAGs at reaction temperature from 40 °C to 70 °C, respectively; LLL-TAG 40 °C, 50 °C, 60 °C and 70 °C, the content of LLL-TAGs at reaction temperature from 40 °C to 70 °C; Conversion 40 °C, 50 °C, 60 °C and 70 °C, the conversion of FAMEs at reaction temperature from 40 °C to 70 °C.

Temperature played an impact role in the interesterification of FAMEs and triacetin. As shown in Figure 3, the content of LLS-TAGs and the conversion of FAMEs were first increased and then decreased as the reaction temperature increased from 40 to 70 °C. High temperature increases the reaction rate as it reduces the viscosity of lipid mixture and therefore increases the substrate and product transfer on the surface or inside the enzyme particles. In addition, higher temperatures will facilitate the removal of the by-product methyl acetate by vacuum and eventually promote the interesterification reaction. However, high temperatures also reduce the stability and half-life of enzymes [20]. The changes in FAMEs conversion and LLS-TAGs content were slight between 50 and 70 °C, which suggested the reaction was not influenced much in this temperature range. The content of LLL-TAGs increased as temperature increased. Since a lower LLL-TAGs content was highly preferred, the optimum temperature was determined to be 50 °C. There were 54.4% of LLS-TAGs and 1.5% of LLL-TAGs in the final product and the FAMEs conversion was 72.2%.

### 2.4. Effects of Enzyme Loading

**Figure 4:**
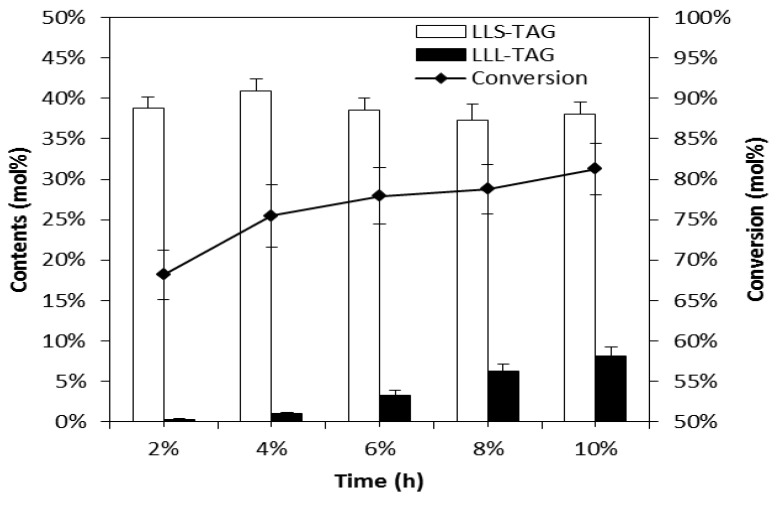
Effects of enzyme loading on interesterification of SLCTs at 24h. The reaction mixture of 9.7 g FAME/triacetin (3:1mol/mol) and different amount of Novozyme 435 was stirred at 50 °C. LLS-TAG, the content of LLS-TAGs; LLL-TAG, the content of LLL-TAGs; Conversion, the conversion of FAMEs.

Generally, there will be higher reaction conversion rate with a higher amount of lipase, until it comes to a saturation point. Therefore, in order to obtain the optimal amount of enzyme for SLCTs synthesis, interesterification of FAMEs with triacetin was performed at 50 °C at the molar ratio of 3:1 using different amounts of Novozyme 435 (2, 4, 6, 8, and 10%) and the SLCTs yields were compared after 24 h. As shown in Figure 4, the conversions of FAMEs and the content of LLL-TAGs increased as the lipase amount increased. To obtain the higher content of LLS-TAGs and lower content of LLL-TAGs, 4% (w/w) enzyme amount was chosen for the subsequent studies.

### 2.5. Scale-Up Synthesis and Purification of SLCTs

Scaled-up production of SLCTs was conducted in a 5 L round-bottom flask. A total of 3.6 kg FAMEs from camellia oil and 0.89 kg of glyceryl triacetin (3:1, molar ratio) and 181 g immobilized lipase (4%, w/w) were added to the reactor vessel and the reaction mixture was stirred at 300 rpm and incubated at 50 °C for 24 h. The scaled-up products were made up of 52.4% of LLS-TAGs and 1.1% of LLL-TAGs, and the FAMEs conversion was 82.4%. The mixture was purified by molecular distillation (MD) to obtain LLS-TAGs, and the triacetin, FAMEs and LSS-TAGs were removed during MD. The composition of the final product was analyzed and the results are shown in Table 1. The final product contained 94.6% of LLS-TAGs and 4.4% of LLL-TAGs. High percentage of LLS-TAGs was successfully collected because of the relatively larger molecular weight of LLS-TAGs. It was verified that the designed reaction system was suitable for large-scale production of pure LLS-TAGs.

**Table 1:** Composition of fractions during preparation of SLCTs (mol%).

Products	LLS-TAGs	LLL-TAGs	Others *
Interesterification product	52.4	1.2	46.4
MD distillates	4.5	0.4	95.1
MD residues	94.6	4.4	1.0

* Include FAMEs, LSS-TAGs and triacetin.

### 2.6. Fatty Acid Composition

The fatty acid (FA) compositions of camellia oil, DAG and the final SLCTs obtained by interesterification were shown in Table 2. The SLCTs had similar composition to the original camellia oil and DAG. It could be concluded that the processes of enzymatic interesterification and molecular distillation had no significant effect on the FA composition.

**Table 2:** FA composition of camellia oil, DAG and SLCTs.

Fatty acid	Fatty acid composition (mol%)		
	Camellia oil	DAG	SLCTs
C2:0	- *	- *	29.16 ± 0.35
C16:0	7.04 ± 0.65	7.05 ± 0.35	5.34 ± 0.43
C18:0	2.72 ± 0.13	2.41 ± 0.19	2.00 ± 0.21
C18:1	80.87 ± 1.74	81.21 ± 1.34	57.29 ± 0.49
C18:2	8.48 ± 0.82	8.45 ± 1.08	5.59 ± 0.63
C18:3	0.46 ± 0.20	0.51 ± 0.16	0.34 ± 0.04
Others	0.43 ± 0.06	0.37 ± 0.13	0.27 ± 0.05
FFA (mg/g)	0.48 ± 0.05	- *	- *

* Not detected.

### 2.7. Plasma Analysis of Fasting Experiment

Plasma TG, TC, LDL-C and HDL-C concentrations in mice 4 h after gastric lavage of different oils were studied, results were shown in Table 3. The TC, LDL-C and HDL-C of all groups showed no significant difference (*p* > 0.05). However, the plasma TG concentrations of all test groups were increased in comparison of blank group. However, the plasma TG levels of the mice dosed with SLCTs and DAG were lower than those with TAG. One reason might be that short and long acyl TAG molecule has lower caloric availability (4.5–6 kcal/g) than LCTs (triacylglycerols with only long-chain acyl residues, 9 kcal/g) [21]. Short-chain fatty acids are also volatile and more rapidly absorbed in the stomach than long-chain fatty acids because of their higher water solubility, smaller molecular size, and shorter chain length. Another reason might be the different mechanism of metabolism of TAG and 1,3-DAG. When oil is injected into the digestive tract, the majority of ingested TAGs are partially hydrolyzed by pancreatic enzymes to 2-monoacylglycerols (2-MAGs) and FAs, then absorbed into small intestinal epithelial cells and immediately resynthesized into TAG molecules by the lipase. On the other hand, 1,3-DAGs are presumed to be hydrolyzed to form 1(or 3)-MAGs and FAs, which are probably absorbed into the mucosa and further hydrolyzed into glycerols and FAs because 1(or 3)-MAGs are not suitable substrates of the lipase in small intestinal epithelial cells [22]. According to the synthetic method and the enzyme used, the major part of SLCTs products obtained was LSL-type (the long chain FAs mainly at the sn-1, 3 position) rather than LLS-type (the long chain FAs mainly at the sn-1, 2 position). Though from the nutritional point of view, the LSL-type might be less nutritional than the LLS-type, from the structure point of view, the LSL-type might be more active in lowing plasma TAG levels. The structure of LSL-type SLCT was similar to that of 1,3-DAG, therefore, it is expected that such a type of SLCT would share the same metabolism mechanism with 1,3-DAG, *i.e.*, the 2-MAG with the short chain in position 2 resulted from the hydrolysis by pancreatic lipase would be digested rapidly and thus lowering the postprandial TAG levels. In addition, research from Porsgaard *et al*. indicated that the intramolecular TAG structure only affected absorption during the first few hours after oil administration, but did not affect the overall absorption of FAs [23]. Thus, the synthesized SLCTs would not affect the absorption of the unsaturated FAs. Moreover, the SLCTs had advantage over DAGs in that they did not contain glycidyl ester. In conclusion, the synthesized SLCTs might act as a potential functional oil that can prevent fat accumulation in the body.

**Table 3:** Plasma analysis of fasting experiment.

Groups	TAG (mmol/L)	SLCTs (mmol/L)	DAG (mmol/L)	Blank (mmol/L)
TC	4.05 ± 0.49	3.92 ± 0.99	3.17 ± 0.61	3.34 ± 0.60
TG	2.64 ± 0.43 ^a^	2.04 ± 0.40 ^a,b^	2.06 ± 0.28 ^a,b^	1.05 ± 0.10
HDL-C	1.54 ± 0.39	1.49 ± 0.47	1.25 ± 0.29	1.39 ± 0.21
LDL-C	10.26 ± 3.13	8.39 ± 3.51	8.11 ± 2.59	9.15 ± 2.55

^a^ The a in an entry denotes significant differences (*p* < 0.05) as compared with control group. ^b^ The b in an entry denotes significant differences (*p* < 0.05) as compared with TAG groups.

## 3. Experimental

### 3.1. Materials

FAMEs (≥98% purity) were produced by methanolysis of camellia oil with methanol catalyzed by sodium methoxide [24]. DAG (95.6% purity) was produced by glycerolysis of camellia oil with glycerol according to the method described by Wang *et al*. [25]. Triacetin (CAS no. 102-76-1) was purchased from Aladdin Chemistry Co. Ltd. (Shanghai, China). Two immobilized lipases (Lipozyme RM IM from *Rhizomucor miehei* and Novozyme 435 from *Candida antarctica*) were purchased from Novozymes A/S (Bagsvaerd, Denmark). A standard mixture with 37 FAMEs (CAS no.113 47885-U) was purchased from Sigma-Aldrich (Shanghai, China). Camellia oil was purchased from a local market. All used chemicals and solvents were either of analytically pure or chromatographically pure grade.

### 3.2. Comparison of Lipase

Two commercial immobilized lipases (Lipozyme RM IM and Novozyme 435) were compared for synthesis of SLCTs. The reaction was performed at molar ratio of FAMEs to triacetin of 2:1, 40 °C of reaction temperature and lipase amount of 2% (w/v) at solvent-free system and water-free system. The reaction flasks were coupled with a vacuum pump and the level of vacuum was kept below 500 Pa.

### 3.3. Synthesis of SLCTs

The interesterification reactions were performed in a solvent-free and water-free system in 25 mL round-bottom flasks. The effects of temperatures (from 40 to 70 °C), substrate molar ratios of FAMEs to triacetin (from 2:1 to 6:1) and lipase loading (from 2% to 8%, by the total weight of substrates) were studied. Flasks were placed in a magnetic stirring apparatus coupled with a vacuum pump. The stirring rate was set at 100 rpm. After given times, products were collected after removal of enzyme. All reactions were performed in triplicate and the results were expressed as the average of molar percentage. 

### 3.4. Analysis of Product

The reaction product was analyzed by Waters 2695 HPLC system (Waters Tech., city, state abbrev, USA) equipped with Waters 2424 evaporative light scattering detector (ELSD). The separation was carried out on a XbridgeTM C18 reverse phase column (5 *μ*m, 250 × 4.6 mm, Waters) according to the reported conditions [8]. The content of the glycerides was calculated based on their area percentages. The peak identification of a single TAG was coupled with mass spectrometry (MS) [26]. The MS conditions were as follows: nebulizer gas, N_2_; P, 0.14 MPa; nebulizer gas flow rate, 10 mL/min; atmospheric pressure chemical ionization (APCI) mode, positive; APCI temperature, 350 °C; Q array (quadrupole array), scan; mass range, m/z 50–1500.

### 3.5. Fatty Acid Composition of SLs Product

The enzyme was removed from the reaction mixture by centrifugation. Sixty microliters of the reaction product was applied to thin-layer chromatography plates (10×20 cm) coated with silica gel G. The developing solvent was petroleum ether/ethyl ether/acetic acid (90:10:1, v/v/v). The plates were air-dried for 30 min, and then sprayed with 0.1% of 2, 7-dichlorofluorescein in methanol and the bands were visualized under ultraviolet light. The TAGs band was scraped off into a 50-mL round-bottomed flask and methylated to FAMEs according to ISO 5509:2000 [27]. The FAMEs were analyzed on an Agilent 7890A GC (Agilent Tech., city, state abbrev, USA) equipped with a capillary column CP-Sil 88 (60 m × 0.25 mm × 0.2 μm, Varian Inc. city, state abbrev, USA) and a flame ionization detector [28]. The temperatures of the injector and the detector were 250 °C and 300 °C, respectively. The column oven was initially held at 140 °C for 5 min and was then programmed to 220 °C at a rate of 4 °C/min and was held isothermally for 15 min. Nitrogen was used as the carrier gas at a head pressure of 0.5 MPa with a flow rate of 1.1 mL/min. The correction factor values of FAMEs were used to obtain the relative content of FAMEs. All datas were expressed as molar percent.

### 3.6. Other Analyses

The free fatty acid (FFA) of the test oils was determined by the alkali titration method [29].

### 3.7. Scale-Up Synthesis and Purification of Product

A scale-up reaction (reaction mixture on a kilogram scale) was performed under optimum conditions in a 5-L round-bottom flask with a glycerol bath and magnetic stirrer. About 3.5 kg of camellia oil-based FAMEs was used in the reactor. The reaction mixture was stirred at 300 rpm and in vacuum condition of 0.02MPa for 24 h. Samples were withdrawn periodically and stored at −20 °C till analysis. Molecular distillation (MD-S80, Handway Technology Inc., Guangzhou, China) was applied to purify the reaction product by removing FAMEs, triacetin and LSS-TAGs. An evaporation temperature of 185 °C, a feed flow rate of 3 g/min, a pressure of 10 Pa and a scraper speed of 200 rpm were used in the process.

### 3.8. Animals and Gastric Lavage Experiments

Forty NIH male mice were purchased from the Guangdong Medical Lab Animal Center (certification: GDMLAC 2007A056) at 5 weeks of age. All mice were kept in an animal room, at a temperature of 20–25 °C, a humidity of 45–55% and a 12 h light/dark cycle. Mice had free access to water and were adapted to the commercial pellet feed (Guangdong Medical Lab Animal Center) for a week and then separated into the following four groups by randomized block design method: Blank, Blank group; TAG, camellia oil group; DAG, DAG group; SLCTs, synthesized SLCTs group. After 18 h fasting, Blank, TAG, DAG and SLCTs group received 0.1 mg per body weight (g) physiological saline, camellia oil, diacylglycerol and synthesized SLCTs by gastric lavage at a dosage of 0.1 mg per body weight (g), respectively. All of the experimental protocols have been approved by the Institutional Animal Ethics Committee of Guangdong Pharmaceutical University (GDPUIAEC no. 200902) which is in a compliance with NIH Guide for the Care and Use of Laboratory Animals (NIH publication no. 85–23, 1985).

### 3.9. Serum Analysis

Four hours after gastric lavage, blood was collected through the orbital sinus from anesthetized animals. Triglycerol (TG), total cholesterol (TC), low density lipoprotein cholesterol (LDL-C) and high density lipoprotein cholesterol (HDL-C) contents were analyzed using chemistry analyzer (AMS-18A, Bei Jing Potion Science & Technology Development Co., LTD., Beijing, China) with a commercial kit (BioSino Bio-technology and Science Inc., Beijing, China).

### 3.10. Statistical Analysis

Data were analyzed using the analysis of variance (ANOVA) procedure of Statistical Product and Service Solutions (SPSS 17). Significant differences were determined by using Duncan’s multiple range tests. Significance of differences were defined at the *p* < 0.05 level. All analytical determinations were carried out in triplicate. The results were reported as the means ± standard deviations (SD).

## 4. Conclusions

In this study, the optimal conditions for the high yield synthesis of SLCTs from camellia oil FAMEs and triacetin were estimated. The synthesis method offered considerable advantages in terms of production of highly pure LLS-TAGs. Based on the optimization of reaction conditions, the reaction mixture of acylglycerols contained 52.4% of SLCTs, and the content of SLCTs in the final product was as high as 94.6%. The effect of the synthesized SLCTs on plasma lipid levels in NIH mice was examined by fasting experiments and the results indicated that the SLCTs could reduce the plasma TAG contents after gastric lavage in NIH mice. In addition, the SLCTs contain unsaturated fatty acids and have lower risk of glycidyl ester formation. It suggested that the synthesized SLCTs might be used as a potent functional oil for suppressing high fat-induced obesity.
